# Effects of preoperative magnesium therapy on arrhythmias and myocardial ischemia during off-pump coronary surgery

**DOI:** 10.4103/1817-1737.53355

**Published:** 2009

**Authors:** Yavuz Beşoğul, Hüseyin Gemalmaz, Recep Aslan

**Affiliations:** *Department of Cardiovascular Surgery, Osmangazi University Medical School and Research Hospital, Eskişehir, Turkey*

**Keywords:** Ischemia and arrhythmia, magnesium effects, off-pump coronary surgery

## Abstract

**BACKGROUND:**

Heart manipulation during off-pump coronary artery bypass surgery may cause hemodynamic instability, and temporary coronary arterial occlusion may lead to myocardial ischemia. To reduce this, perioperative β-blocking agents or calcium antagonists can be administrated. The effects of perioperative administration of magnesium on myocardial function were studied in patients undergoing coronary artery bypass grafting.

**OBJECTIVE:**

The aim of the study was to evaluate the effects of preoperative magnesium administration on perioperative hemodynamia, ventricular arrhythmias and myocardial protection.

**MATERIALS AND METHODS:**

We reviewed 2 groups of patients undergoing off-pump coronary artery bypass surgery – 24 patients (control group) that had not received preoperative intravenous infusion of magnesium and 23 patients (treatment group) that had received preoperative intravenous magnesium sulfate.

**RESULTS:**

The results demonstrated that it had reduced the heart rate, changes of ST segments, the need of β-blocking agents and the use of intra-operative intra-aortic balloon pump and the inotropic usage.

**CONCLUSION:**

This treatment may provide hemodynamic optimization during off-pump coronary artery bypass.

The performance of off-pump coronary artery bypass graft surgery (OPCAB) has been extensive because of the advantage of avoiding bypass-related cardiopulmonary complications.[1–5] OPCAB allows continuous perfusion ofa beating heart, but it is with temporary occlusion of the target artery and, possibly, hemodynamic instability with subsequent reduction in coronary flow.[6] The consequences of this temporary coronary occlusion may be relatively insignificant or may lead to myocardial ischemia (MI), severe heart failure and, ultimately, cardiac arrest. [7] Magnesium is considered to be a naturally physiologic calcium blocker. Animal models suggest that magnesium supplementation before reperfusion reduces infarct size.[8] Until recently, only small studies have indicated there may be reduction of hemodynamic instability due to heart manipulation during OPCAB if preoperative magnesium infusion was added to therapy. The purpose of this study was to assess the effect of preoperative infusion of magnesium 24 hours prior to off-pump surgery on hemodynamic instability, arrhythmias and MI and to find factors that can influence the efficacy of this treatment.

## Materials and Methods

The charts of 47 patients that had undergone off-pump coronary artery bypass surgery were retrospectively reviewed. Only elective operations were included in the study. Patients that were in hemodynamically unstable condition or showed evidence of acute ischemia before surgery were excluded from the study. Demographic data, perioperative hemodynamic data and postoperative arrhythmias were recorded and compared among patients that did and did not receive preoperative magnesium administration. The first 24 patients did not receive the prophylactic treatment, whereas the next 23 patients were treated with magnesium preoperatively. Patients in the magnesium-treated group received 1.5 mg (=12 mEq) of magnesium sulfate, infused daily for 2 days prior to surgery.

### Operative technique

Anesthesia was induced with fentanyl and sodium thiopental and maintained with fentanyl. Muscular relaxation was obtained with pancuronium bromide. Left internal mammary artery (LIMA) was harvested after routine median sternotomy. After systemic heparinization (1mg/kg), the pericardium was opened. A silastic tape was placed around the left anterior descending (LAD) to produce proximal coronary occlusion, and epicardial stabilizing device was placed.The LIMA-to-LAD anastomosis was completed. Then saphenous vein graft was anastomosed to the right coronary artery with the silastic tape and the epicardial stabilizing device. The partial cross clamp was applied to the aorta for placement of the proximal anastomoses.

### Statistical analysis

Tests of normality and Student *t* tests were used to compare the preoperative characteristics of the patients. Perioperative heart rate and mean arterial pressure were compared using Mann-Whitney U test, and ST changes were analyzed using Fisher exact test. The other intra-operative variables were compared using Chi-square tests. *P* values less than .001 were considered significant.

## Results

The clinical characteristics of the groups were similar, and there were no significant differences [Table 1]. Intra-operative data are shown in Table 2. The average of ejection fraction was lower in the treatment group. Comparison of the intra-operative findings between the 2 groups showed that the heart rate, changes of ST segments, and the need of β-blocking agents were lower in the magnesium group as compared to the no-treatment group (*P* <.05). Otherwise, the use of intra-operative intra-aortic balloon pump and the inotropic usage were low, but they were insignificant statistically because the variables were fewer. Ventricular tachyarrhythmias and the need of defibrillation were observed to be higher in the no-treatment group as compared to the magnesium group.

**Table 1:** Clinical characteristics of the groups

Characteristics	Preoperative treatment (n = 23) (%)	No treatment (n = 24) (%)
Male	19 (82.6)	21 (87.5)
Female	4 (17.4)	3 (12.5)
Age (Years)	58.8 ± 12.3	61.4 ± 7.04
1-vessel bypass	13 (56.6)	18 (75)
2-vessel bypass	9 (39.1)	6 (25)
3-vessel bypass	1 (4.3)	0
Mean ejection fraction	35.1 ± 9.35	41.7 ± 11.1
Diabetes mellitus	7 (30.4)	6 (25)
Chronic obstructive pulmonary disease	5 (21.7)	6 (25)
Preoperative β-blockers	0	0
Preoperative digital usage	0	2 (4.2)
Previous myocardial infarction	18 (78.2)	17 (70.8)
Preoperative intra-aortic ballon support	7 (30.4)	5 (20.8)
Preoperative inotropic usage	0	1 (4)

There were no statistically significant differences in the clinical characteristics between the two groups (*P* > .05)

**Table 2:** Intra-operative data of the groups

Intra-operative data	Preoperative treatment (n = 23) (%)	No treatment (n = 24) (%)	*P* value
Heart rate	77.39 (±11.26)	90.95 (±10.66)	<0.001
Mean arterial pressure	71 (±5.63)	67.7 (±5.51)	0.053
Elevation of ST segment	5 (21.7)	12 (50)	0.034
Inotropic agents	3 (13)	8 (33.3)	0.49
β-blockers	1 (4.3)	11 (45.8)	0.003
Calcium antagonists	0	0	0
Perioperative intra-aortic balloon pump	0	5 (20.8)	0.050
Ventricular arrhythmias	1 (4.3)	6 (25)	0.097
Defibrillation	1 (4.3)	3 (12.5)	0.60
Temporary pacing	0	0	0
Postoperative MI	0	0	0

Data are presented as number of observations, with percentages in parenthesis; or as mean ±SD. (*P* values .05 and .001 are significant.)

Serum levels of magnesium were in the normal range (1.5-2.6 mg/dL) in all patients of both the groups before operation. These were minimally increased in 3 patients in the magnesium-treated group, but these increases were not significant. No patient required temporary pacing and no patient died in the early postoperative period.

## Discussion

This is the first report to demonstrate that preoperative magnesium administration provides hemodynamic optimization during off-pump coronary bypass. Magnesium is known as a natural calcium antagonist, and calcium mediates the major mechanism of ischemia-reperfusion myocardial damage.[9–10] Animal models suggest that magnesium supplementation before reperfusion reduces infarct size (8). Guo *et al.*[11] believed that high magnesium levels decrease reactivity of vessels, thus improving the blood flow through the coronary vessels. Recent reports stress the importance of the therapeutic effects of magnesium in acute heart ischemia[12] Ravn *et al.*[13] reported that intravenous administration of magnesium resulted in at least 50% reduction in infarct size.

During the period of distal anastomosis in the OPCAB surgery, there is no turning backflow. The consequences of this temporary coronary occlusion may lead to severe heart failure and, ultimately, cardiac arrest. In addition, the ST segments may become severely elevated or depressed. Once the anastomosis is complete, the silastic tape is removed, coronary flow is reestablished and both cardiac index and ST segment changes should improve.[7] Data from both observational and randomized trials suggest that Q-wave and non-Q-wave infarction appear in the OPCAB patients. Other parameters of myocardial injury, such as troponin I, creatine phosphokinase of muscle band (CPK-MB) and myoglobin, are significantly lower, because of the short duration of the temporary occlusion.[14–19] To reduce the myocardial ischemia, it is imperative to improve myocardial oxygen balance by reducing the oxygen consumption through a decrease in heart rate and contractility, as well as through decrease in the occurrence of arrhythmias. Perioperative β-blocking agents and calcium antagonists can be used for the management of hemodynamic stability.[20] Balser[21] reported that the small-dose postoperative magnesium supplementation reduced the incidence of ventricular arrhythmia. Caspi *et al.*[22] showed that perioperative administration of magnesium contributed to better myocardial recovery and fewer postoperative ventricular tachyarrhythmias. Maintaining an adequate mean arterial pressure (MAP) is the most important maneuver as far as decreasing myocardial ischemia is concerned. MAP of ≥70 mm Hg is safe and may be achieved with the help of positioning[23] Elevation of extracellular magnesium levels reduces arteriolar tone and tension in a wide variety of arteries and potentiates the dilating effects of some endogenous (adenosine, potassium and some prostaglandins) and exogenous (isoproterenol, nitroprusside) vasodilators. As a result, magnesium can mildly reduce systolic blood pressure, thereby unloading the ischemic ventricle. Magnesium reduces systemic and pulmonary vascular resistance, with a concomitant decrease in blood pressure and a slight increase in the cardiac index[24]

In the present study, it has been observed that preoperative magnesium administration for prophylaxis reduced the heart rate, myocardial infarction, the needs of β-blocker agents, the extent of intra-operative intra-aortic balloon pump support required, and the incidence of ventricular arrhythmias during the period of distal anastomosis in the OPCAB. We believe that this treatment is effective in providing hemodynamic stability and efficacious MAP in the OPCAB surgery. But larger serial studies are needed to investigate this, and the parameters of myocardial injury may be explored during the coronary artery occlusion for anastomosis.
